# *Prathapanius fortis*, a new genus and new species of Galerucinae from Ecuador (Coleoptera, Chrysomelidae)

**DOI:** 10.3897/zookeys.968.54228

**Published:** 2020-09-16

**Authors:** Keezhpattillam Viswajyothi, Shawn M. Clark

**Affiliations:** 1 Monte L. Bean Life Science Museum, Brigham Young University, Provo, Utah 84602, USA Brigham Young University Provo United States of America; 2 Kerala Agricultural University, Department of Agricultural Entomology, College of Agriculture, Vellayani, Trivandrum, Kerala, 695522, India Kerala Agricultural University Trivandrum India

**Keywords:** Diabroticina, Diabroticites, leaf beetles, Luperini, Neotropics, sexual dimorphism

## Abstract

*Prathapanius* Viswajyothi & Clark, **gen. nov.**, is described and illustrated. The genus is placed in the chrysomelid section Diabroticites Chapuis (subtribe Diabroticina Chapuis, tribe Luperini Chapuis, subfamily Galerucinae Latreille). It is monobasic, containing *Prathapanius fortis* Viswajyothi & Clark, **sp. nov.**, from Ecuador. The new genus is briefly compared with *Acalymma* Barber, *Isotes* Weise, and *Zischkaita* Bechyné.

## Introduction

Over the last half century, the prevailing classification of the chrysomelid subfamily Galerucinae Latreille has largely followed the catalogues of Wilcox (1971, 1972, 1973), and it is reflected in the subsequent list of genera by Seeno and Wilcox (1982). However, several modifications have occurred subsequent to those catalogues, with perhaps the most important being the combining of the former subfamily Alticinae Newman with Galerucinae. This taxonomic change was due to apparent paraphyly, as demonstrated in numerous studies (e.g., Gillespie et al. 2008). However, this union is resisted in some very important works, such as the Catalogue of Palaearctic Coleoptera by Löbl and Smetana (2010). Indeed, some studies recover both Alticinae and Galerucinae as monophyletic clades (e.g., Nie et al. 2020). Some recent studies recognized an enlarged Galerucinae, with just two tribes (Galerucini Latreille and Alticini Newman), and they treat the tribes of the old Galerucinae as subtribes (e.g., Duckett et al. 2004; Nie et al. 2018). Such an approach largely defeats the logic for combining Alticinae with Galerucinae. That is, if the two groups are monophyletic, then they both might as well be treated at subfamily rank.

In the present study, we follow a middle-of-road approach, provisionally accepting the combining of Alticinae with Galerucinae, but retaining all the tribes of the former Galerucinae at tribal rank. This approach was also followed in works such as by Riley et al. (2002, 2003). Hence, we recognize six galerucine tribes (Oidini Chapuis, Galerucini, Metacyclini Chapuis, Hylaspini Chapuis, Luperini Gistel, and Alticini). The tribes are subdivided into subtribes (with -ina endings), which are further subdivided into sections (-ites endings). Oddly, the subtribal rank is sometimes omitted, the tribes being directly divided into sections. For the most part, the tribes are fairly well differentiated from each other, based on morphology. However, some of the lower categories, especially the sections, are very poorly characterized, and their validity is doubtful.

Following Seeno and Wilcox (1982), three non-alticine tribes of Galerucinae occur in the New World (Galerucini, Metacyclini, and Luperini). These include nearly 1900 named species distributed in about 100 named genera. The Luperini are especially pertinent to the present study. In these beetles, the aedeagi lack prominent basal spurs (present in Galerucini and Metacyclini), the antennae usually extend from near the middle level of the eyes or even higher (below the middle level of the eyes in most Galerucini and many Metacyclini), and the larvae are root feeders (leaf feeders in Galerucini; unknown in most Metacyclini, but leaf feeders in at least one genus). Within the Luperini, the subtribe Diabroticina Chapuis is characterized by the absence of a rectangular lobe at the apex of the male abdomen, and, within the Diabroticina, the section Diabroticites Chapuis is characterized by “bifid” tarsal claws, that is, the inner appendage of each claw is rather narrow and acutely pointed (in most species in the other sections of the subtribe, the inner appendage is “appendiculate,” that is, apically blunt (Wilcox 1965).

Phylogenetic studies do not entirely support the above classification, even at the level of tribe (Gillespie et al. 2003, 2004, 2008; Duckett et al. 2004; Nie et al. 2020). However, in one of the most extensive studies dealing with galerucine phylogeny, the subtribe Diabroticina was recovered as monophyletic. In the same study, the section Diabroticites was largely also recovered as monophyletic, except that some of the species of *Gynandrobrotica* Bechyné (currently Diabroticites) were nested within the section Cerotomites Chapuis. Despite fundamental doubts regarding some of the groups, the prevalent classification is still useful for organizing the species, and it will remain so until changes to the classification are formalized.

In anticipation of the eventual publication of a key to the New World genera of Galerucinae (exclusive of Alticini), we have examined examples of nearly every genus of the subtribe Diabroticina. Beyond studying authoritative determined material, we have also attempted identification of many thousands of previously unidentified specimens. We discovered specimens that clearly do not match any of the described genera, and we herein propose a new genus to accommodate them. The single included species has remarkably modified legs in the male.

Strange morphological modifications are common in the chrysomelid subfamily Galerucinae. Although some are present in females, most are secondary sexual characters present only in males. They involve the head, antennae, pronotum, elytra, legs, etc. In many instances, their function is unknown. However, some of them may be related to mate recognition, pheromone dispersal and detection, sound production, or grasping during mating (Mohamedsaid and Furth 2011). The sexually dimorphic characters within the section Diabroticites were evaluated by Prado (2012), and they were found to be only partially useful in differentiating genera. The genus described herein is most easily recognized by the male modifications but is distinguished by other characters as well.

## Materials and methods

All specimens studied were adults. Genitalia extractions and preservation largely followed the methodology described by Smith (1979). Specimens were examined using Wild M5A and Olympus SZ61 stereoscopes. Measurements were made using an ocular scale in an Olympus WHSZ10X-H/22 eyepiece on an Olympus SZ61 stereoscopic microscope. Microphotography employed an Olympus SzX12 dissecting microscope equipped with an MTI 3CCD camera and an Olympus MVX10 dissecting microscope equipped with an Olympus DP70 camera. Image montage employed Olympus cellSens software. Images were processed with Adobe Photoshop 2020 (21.1.2). Descriptive terminology largely follows Konstantinov (1998).

Specimens are deposited in the Monte L. Bean Life Science Museum, Brigham Young University (Provo, Utah, U.S.A.; BYUC), Museo de Zoología de la Pontificia Universidad Católica del Ecuador (Quito, Ecuador; QCAZ), and Travancore Insect Collection, Kerala Agricultural University (Vellayani, Kerala, India; TIC). Label data from the specimens are presented verbatim; a backwards slash (\) indicates a new line on a label.

### Prathapanius

Taxon classificationAnimaliaColeopteraChrysomelidae

Viswajyothi & Clark gen. nov.

2A6BA0DF-ED77-59C3-BC6C-7713AB1043C6

http://zoobank.org/47D955A8-44F0-47DC-AD4D-8D89A98F0DC0

**Figure 1.**
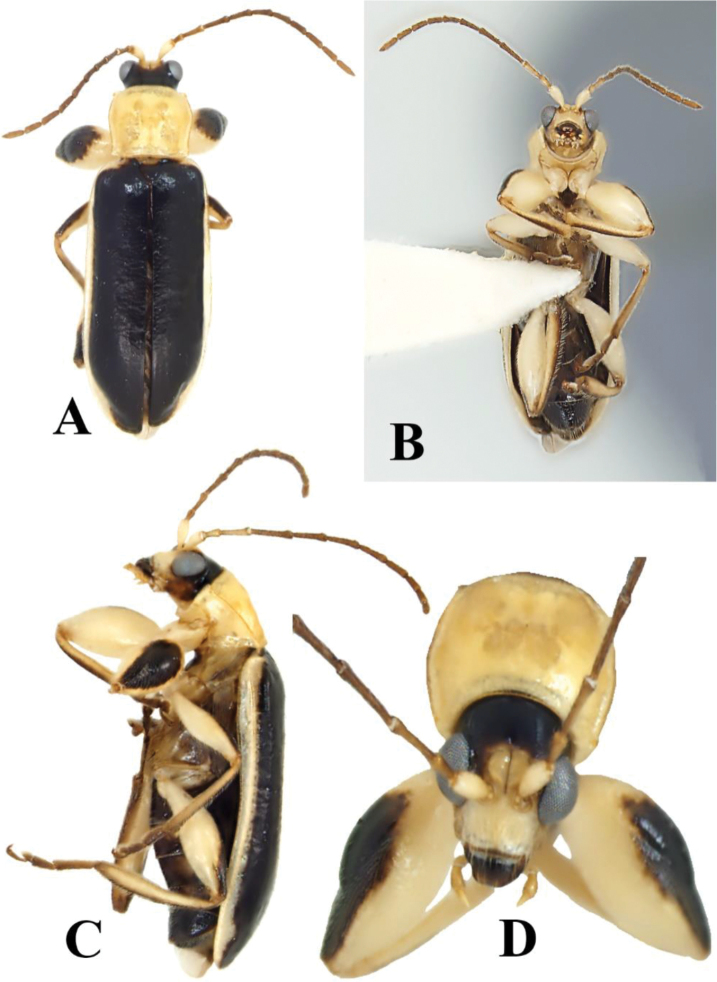
*Prathapanius fortis* sp. nov., holotype. **A** Dorsal aspect **B** ventral aspect **C** lateral aspect **D** anterior aspect.

**Figure 2.**
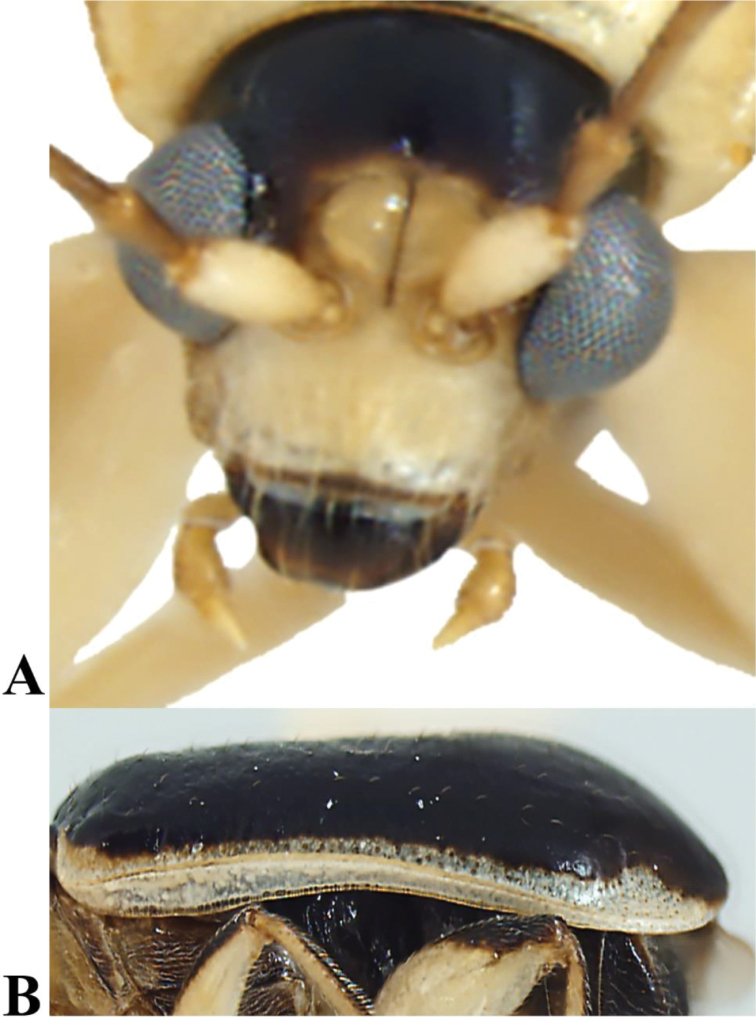
*Prathapanius fortis* sp. nov., holotype. **A** Anterior aspect, close up **B** epipleuron.

**Figure 3.**
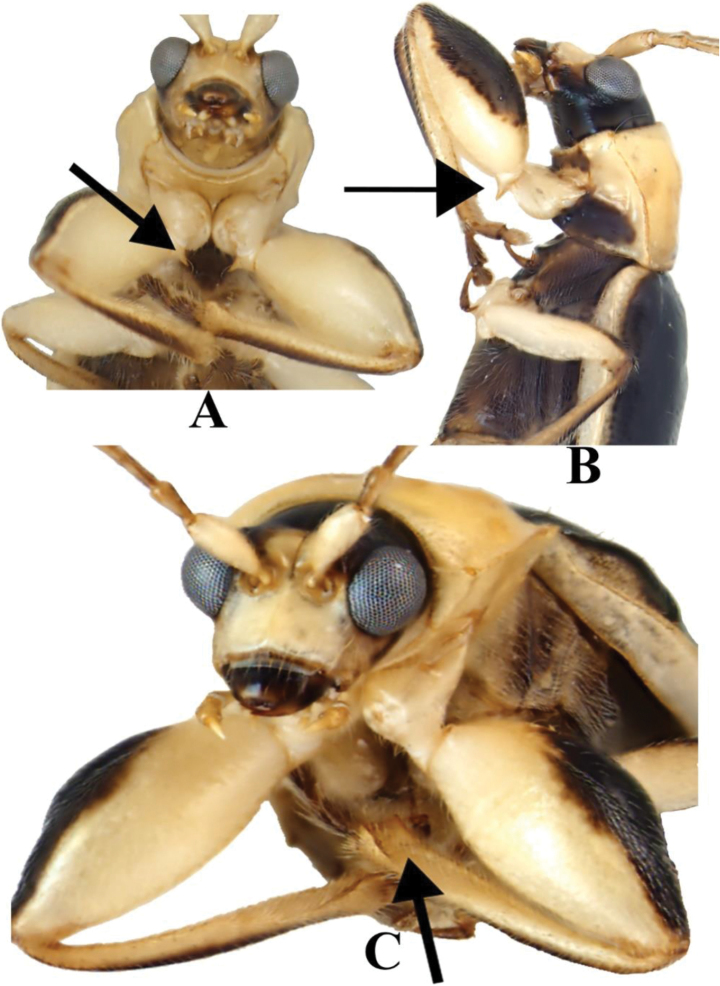
*Prathapanius fortis* sp. nov. **A** Holotype, arrow pointing to spine on front trochanter **B** paratype, arrow pointing to spine on front trochanter **C** paratype, arrow pointing to protibial ridges.

**Figure 4.**
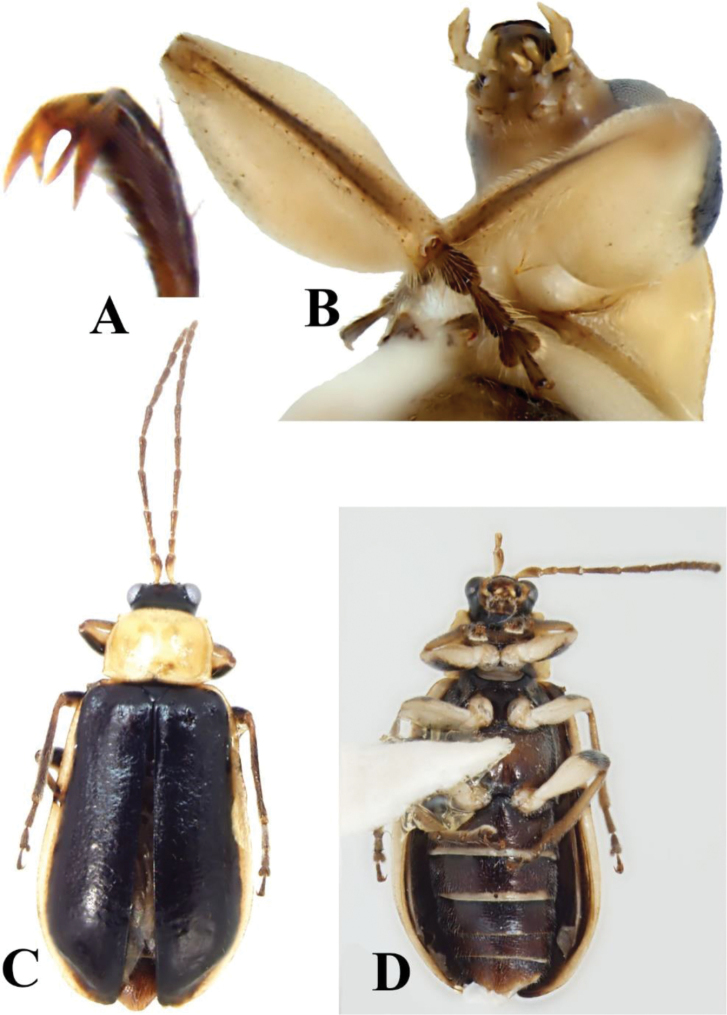
*Prathapanius fortis* sp. nov., paratypes. **A** Bifid claws **B** male, prothoracic leg and maxillary palpi **C** female, dorsal aspect **D** female, ventral aspect.

**Figure 5.**
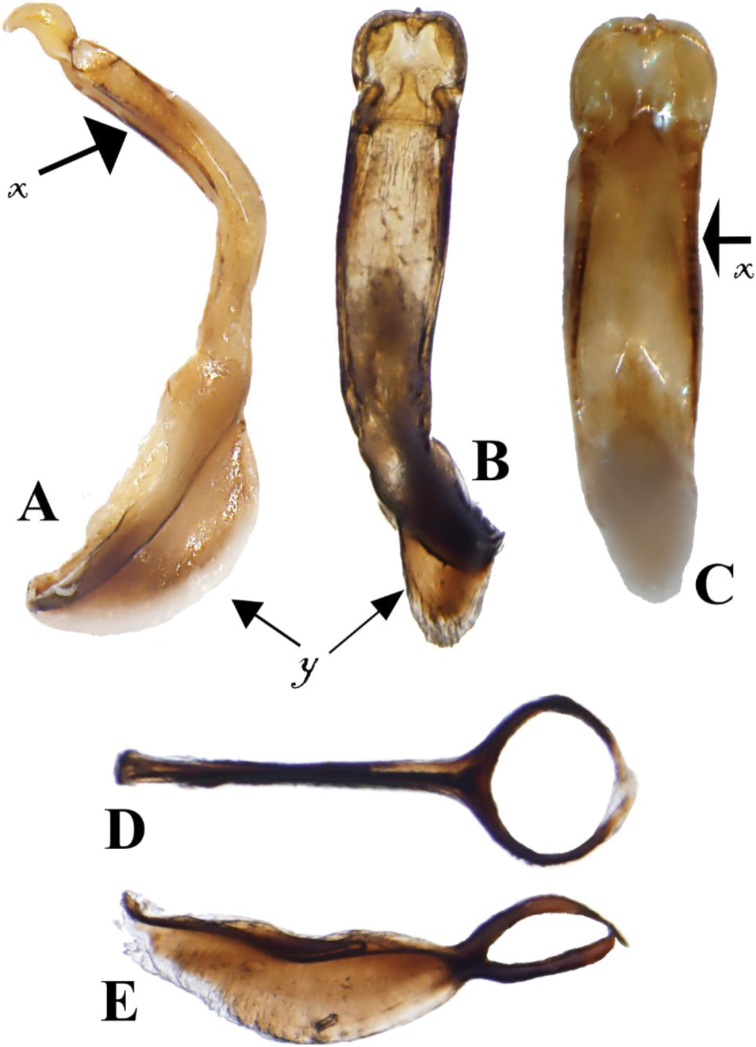
*Prathapanius fortis* sp. nov., paratype. **A** Median lobe of aedeagus, lateral aspect **B** median lobe of aedeagus, dorsal aspect **C** median lobe of aedeagus, ventral aspect **D** tegmen, dorsal aspect **E** tegmen, lateral aspect, **x** = ventral carina on either side of depression, **y** = fin-like structure.

**Figure 6.**
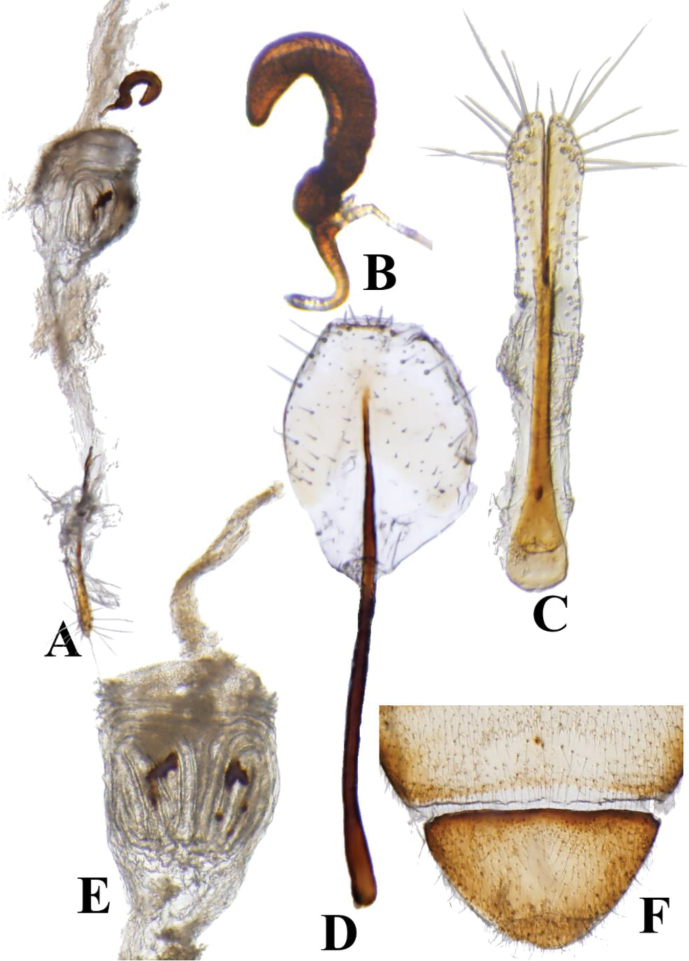
*Prathapanius fortis* sp. nov., paratype. **A** Female genitalia **B** spermatheca **C** vaginal palpi **D** tignum **E** bursa copulatrix **F** fifth female ventrite.

Figs 1 , 2 , 3 , 4 , 5 , 6

#### Diagnosis.

This genus is easily recognized by the remarkable male characters. The front femora are enormously enlarged (Figs 1, 3, 4B), and each front trochanter is posteriorly produced to form a spine-like process (Figs 1B, 3A, B). These two features are not both present in any other known New World galerucine genus. Beyond the male characteristics, the genal space (distance from the eye to the base of exposed mandible) is extremely large (equal to about half the maximum diameter of the eye). This condition is also present in genera such as *Gynandrobrotica* and *Isotes* Weise, but, unlike *Prathapanius*, they lack erect setae on the elytra.

Using the key to genera by Smith and Lawrence (1967), this new genus would be identified as *Synbrotica* Bechyné (couplet 4), which is a junior synonym of *Isotes*. Similarly, in the key by Derunkov et al. (2015), it would be identified as *Isotes* (couplet 4). In both instances, *Prathapanius* may be distinguished by above-mentioned characters.

#### Description.

Body narrow, oblong (Fig. 1A). Antennal calli well developed, raised, convex, triangular, slightly longer than wide; antennal fossae slightly ventral to middle level of eyes (Fig. 2A); genal space large, equal to at least half maximum diameter of eye; antennae slender, filiform, uniformly setose throughout length; apical maxillary palpomere slender, much longer than broad. Pronotum lacking bead or fringe of setae along anterior margin; lateral margin carinate, slightly sinuate, with evenly spaced setae along entire length; posterior margin with bead in lateral portions, without bead mesally, with a few short setae, without well-developed setal fringe. Elytra with numerous well-separated, erect setae, somewhat arranged in longitudinal rows; humeral calli well developed; basal calli poorly developed, not delimited behind by depression; epipleuron parallel-sided in basal half, gradually, slightly narrowed in distal half, disappearing before apex (Fig. 2B). Procoxal cavities open behind; protrochanter of male with sharp, posterior projection (Figs 1B, 3A, B); profemur of male greatly enlarged (Figs 1, 3, 4B); metafemur of male enlarged, but not as much as profemur (Fig. 1B, C); all tibiae with dorsal ridge; tibial spur present in mesothoracic leg of male, in all legs of female; tarsal claws bifid, with inner appendage long and of nearly same thickness as outer appendage or slightly narrow (Fig. 4A); front and middle basitarsi with denser ventral setation than hind basitarsus. Apical margin of last abdominal ventrite lacking lobe or depression, slightly sinuate in male, evenly rounded in female (Fig. 6F). Median lobe of aedeagus with dorsal fin-like structure in basal third (Fig. 5A); basal spurs absent. Tegmen with fin-like structure, similar to that on median aedeagal lobe (Fig. 5E).

#### Type species.

*Prathapanius fortis* Viswajyothi & Clark, sp. nov.

#### Etymology.

The name of this new genus honors K. D. Prathapan, renowned chrysomelid systematist and advisor to the senior author. The enormous front femora of the males are comparable to the strong personality and scientific prowess of Dr Prathapan. The genus name should be treated as a masculine noun.

#### Comments.

This genus clearly belongs in the galerucine section Diabroticites (Luperini, Diabroticina), as evidenced by the absence of basal spurs on the aedeagus, the absence of a rectangular lobe at the apex of the male abdomen, and the presence of bifid tarsal claws. However, the relationships within this section are debatable. The short setae along the lateral margin of the pronotum, as well as the longer setae on the elytral disc, suggest a relationship to *Acalymma* Barber and *Zischkaita* Bechyné. However, these genera have short genae. The large genae of *Prathapanius* suggest a relationship with *Isotes*. This is reinforced by the fact that some (but not all) species of *Isotes* have a small keel on the aedeagus, although not nearly as large as the dorsal fin of *Prathapanius* (Rodrigues and Mermudes 2015). However, *Isotes* lacks the above-mentioned elytral setae. As already mentioned, the remarkable features of the male legs and genitalia of *Prathapanius* are unlike any other known genus of Neotropical Galerucinae.

This new genus currently includes a single species. However, future investigation may prove that *Zischkaita serrana* Moura also belongs here, although it is a much less slender beetle. The male front legs of *Z. serrana* exhibit modifications very similar to those of *Prathapanius*, except that the femoral enlargement is not nearly as dramatic (Moura 2003). The original description of *Z. serrana* does not mention the genal length or genitalia, and we have not examined specimens.

### Prathapanius fortis

Taxon classificationAnimaliaColeopteraChrysomelidae

Viswajyothi & Clark sp. nov.

E28EEAAF-A969-5AF4-907A-7B77D9DB18E4

http://zoobank.org/23E4C884-A7A3-4E17-9CF8-25EC1FC85D2D

Figs 1 , 2 , 3 , 4 , 5 , 6

#### Description of holotype (male).

Body narrow, oblong, 5.1 mm long, 1.6 mm wide (Fig. 1A–C). Surface shiny. Head, venter, antennae, and legs bicolored; pronotum entirely pale; elytra black with lateral areas pale. Numerous semi-erect setae present on elytra. Prothoracic legs greatly modified (Figs 1, 3, 4B).

***Vertex*** black; antennal calli, frontal ridge, and anterofrontal region entirely pale (Figs 1D, 2A); area behind eyes transitioning from pale yellow to brown to black. Vertex glabrous, impunctate, shiny; coronal suture deep anteriorly, continuing as shallow, narrow, distinct suture posteriorly. Eyes oval, 1.2 times as long as wide, finely faceted, narrowly separated from antennal fossae; interocular distance, in anterior aspect, equal to almost half diameter of head across eyes. Antennal calli glabrous, well developed, raised above level of vertex and frontal ridge when observed in lateral aspect, distinctly convex, triangular, slightly longer than wide, broadly contiguous with each other along meson, vaguely extending to posterior third of antennal fossae, attaining less distinct frontal ridge; midfrontal sulcus narrow, distinct; supracallinal sulcus indistinct, represented by shallow, wide depression. Orbit distinct, 0.3 times as wide as antennal calli; orbital sulcus obsolete, with several small setae nearby; supraorbital sulcus distinct; supraorbital pore distinct, with long seta, with a few smaller setae nearby. Antennal fossae oval, extending from near middle level of eye to near lower fourth of eye; fossal diameter equal to 3.5 times distance between eye and fossa, 1.8 times distance between fossae. Frontal ridge narrow, poorly developed, not separating antennal calli, fusing with antennal calli slightly above mid-level of antennal calli; anterofrontal ridge narrow, obsolete, depressed to flat, with rather long setae; frontoclypeal suture brown, with transverse row of setae. Clypeus pale, slightly wider than labrum, almost as wide as distance between lateral margins of antennal fossae. Gena glabrous, very large, with distance from edge of eye to base of exposed mandible equal to almost half maximum diameter of eye (Figs 1D, 2A, 3B, C); postgena with sparse, long setae.

***Antennae*** slender, filiform, extending to about middle of elytra, entirely covered with short setae, with a few longer setae present. Basal antennomere entirely pale yellow; second and third antennomeres pale yellow ventrally, dark brown dorsally; fourth through eleventh antennomeres dark brown. First antennomere widest, inflated, club-shaped; length ratios of antennomeres (comparisons to antennomere 1): 1.0, 0.5, 1.0, 1.3, 1.1, 1.0, 0.8, 0.8, 0.8, 0.7, 0.8; length to width ratios: 2.5, 2.5, 5.0, 7.0, 5.5, 5.5, 5.5, 4.5, 4.0, 4.0, 4.5; distal conical portion of eleventh antennomere separated by shallow groove, giving false appearance of additional antennomere.

***Labrum*** semicircular, nearly twice as wide as long, with anterior margin almost entire; color brownish black; six setae present, arranged in transverse row, three on either side of middle, arising from basal third of labrum, extending slightly beyond distal margin; distal margin with a few additional, minute setae, arranged in transverse row. Mandibles brown, tridentate, slightly exposed beyond edges of labrum in anterior aspect. Maxillary palpi pale, slender, with setae arising laterally; penultimate palpomere more setose than others, largest in length and width (Figs 1D, 4B); apical palpomere slender, conical. Labial palpi short, pale; gula pale.

***Pronotum*** entirely pale; shape nearly quadrate, slightly wider just anterior to middle, with slightly sinuate lateral margins; greatest width 1.3 times more than that of head across eyes; surface glabrous, shiny, alutaceous, obsoletely punctate, with scattered punctures on disc visible only on close examination; distinct mesal depression present in basal half; lateral bead equipped with short setae along entire length; anterolateral seta-bearing pore obtusely angulate; posterolateral seta-bearing pore acutely angulate. Scutellum black, glabrous, impunctate, shiny, with alutaceous microsculpture.

***Elytra*** black, with lateral areas and epipleura uniformly pale (Fig. 1A, C). Length 3.9 mm; width across humeri 1.3 times as great as maximum width of pronotum. Humeri well developed; basal calli poorly developed, not delimited behind by depression. Discal surface alutaceous, with distinct rows of slightly posteriorly inclined setae; punctures indistinct. Epipleura oblique, slanting ventromesally from epipleural fold towards body; sides parallel in basal half, gradually, slightly narrowed in distal half, disappearing before apex (Figs 1C, 2B).

***Ventral*** areas of prothorax pale, glabrous (Figs 1B, 3A); distance from procoxa to anterior edge of thorax subequal to length of second antennomere; anterior margin with fringe of long setae; posterior prosternal process narrow, short, not separating coxae; procoxal cavities open behind. Mesothorax pale yellow to brown; setation of mesepimeron and mesepisternum dense, similar to that of metepisternum and lateral portion of metasternum; mesosternum nearly glabrous. Metathorax pale yellow to brown; length about equal to combined lengths of first and second abdominal ventrites; pubescence dense on episternum and lateral areas of sternum, slightly sparser and longer in mesal area of sternum. Abdomen brownish black; pubescence composed of long setae, mostly evenly distributed, although distinctly denser on terminal ventrite; fifth ventrite longer than fourth ventrite, shorter than third and fourth ventrites combined; last ventrite slightly sinuate along posterior margin, without depression or distinct lobe.

***Legs*** mostly pale (Fig. 1C); coxae and trochanters entirely pale; femora pale with brownish black dorsal area extending from basal fourth to apex in front leg, from basal half to apex in middle and hind legs; tibiae yellow, with dorsal area darker brown; tarsi dark brown. All femora covered with setae, which are short and denser dorsally, longer and sparser ventrally and ventrolaterally; all tibiae with dorsal ridge; third tarsomere of all legs shorter than first, second, or terminal tarsomere (Fig. 4B); terminal tarsomere of all legs more slender than first, second, or third tarsomeres, nearly twice as long as third tarsomere; tarsal claws bifid; inner appendages of all claws converging towards each other, diverging away from outer appendages, as slender as outer appendages or slightly narrower, 0.6 times as long as outer appendages (Fig. 4A). Prothoracic legs with distal setae of femora, tibiae, and tarsomeres longer in comparison with those of middle and hind legs (Fig. 3B); procoxae prominent, subconical (Figs 1B, 3A, B), with patches of medium sized setae; protrochanter with sharp, spine-like, posterior projection; profemur spindle-shaped, greatly inflated (Figs 1, 3, 4B), uniformly covered by subappressed setae that are longer ventrally than dorsally; protibiae flattened and broadened in distal fourth, with two distinct, brown carinae facing femur in distal fifth, apically forming lateral projections (Fig. 3C); protibial setae denser and longer than those of femora; tibial spur absent; basitarsus short, cylindrical, twice as long as wide, almost as long as second tarsomere, distinctly wider than second tarsomere; second tarsomere conical; basitarsal setae forming ventral adhesive pad. Mesocoxae shiny, globular, slightly pubescent, narrowly separated from each other by pale mesosternal process; mesotrochanters pale, concolorous with femora; mesofemora slender; mesotibiae densely covered with short setae, darker than femora, darker dorsally than ventrally; tibial spur present, tiny, more or less hidden among nearby setae; basitarsus thicker and 1.5 times longer than second tarsomere; basitarsal setae forming ventral adhesive pad. Metacoxae transverse; metatrochanters pale, concolorous with femora; metafemora enlarged, but much more slender than profemora; metatibiae thicker in distal two-thirds than in basal third, with setae shorter on surface opposing (ventral to) femur than on opposite (dorsal) surface, with setae longer in distal half than basal half; tibial spur absent; hind basitarsus about as wide as and twice as long as second tarsomere.

Median lobe of aedeagus (dorsal aspect) broad in distal tenth, shallowly incised at distal tenth, gradually narrowing from behind incisure to near mid-length (at mid-length, 0.6 times maximum width), slightly broadening from mid-length towards base; apex truncate in dorsal aspect, with small, median, knob-like projection (Fig. 5B); orificial surface between lateral incisures, with triangular depression, bordered by distinct lateral carina; in lateral aspect, median lobe strongly bent near middle (Fig. 5A); basal third dorsally expanded to form thin, semicircular, fin-like structure (Fig. 5A); in ventral aspect, median lobe with long, distinct channel extending from median bend to distal tenth, laterally bordered by distinct carina (Fig. 5A, C); basal opening ventral, occupying basal third of median lobe length, almost three-fourths as long as dorsal fin. Tegmen with two slender, curved, lateral arms beginning at distal fourth, forming circle in dorsal aspect (Fig. 5D); basal stem ventrally with fin-like structure, 0.4 times wider than long, similar to fin on median lobe (Fig. 5E).

#### Variation.

The body varies from 4.8 to 5.6 mm long. The head may be almost entirely pale, with only a small brown marking on the posteromedial area of the vertex. This varies to an almost entirely black head, with only parts of the antennal calli and anterofrontal region pale. In some specimens, the region below the eyes and the antennal fossae are pale, while the frontal ridge and anterofrontal ridge are brownish black. In others, the frontal ridge is pale, the antennal calli and the entire area beyond the eyes and antennal fossae are brown, while the rest of the head is black. In some specimens, the dorsal surface of basal antennomere is distally or entirely brown. Rather than entirely pale, the pronotum may have a brownish black, irregularly shaped macula (Fig. 4C). The ventral areas of prothorax may be partially black (Fig. 4D). The venter of some specimens is entirely pale yellow, while that of others is largely brownish but with the area immediately anterior to each mesocoxa and the posterior margin of the abdominal ventrites paler (Figs 1B, 4D). Apparently, the elytral setae are easily abraded, and they are therefore not abundantly present in some specimens.

#### Female.

Although lacking the odd modifications of the legs, females are much like males. The antennomere length ratios are 1.0, 0.6, 1.0, 1.5, 1.1, 1.1, 1.0, 0.8, 0.8, 0.8, and 1.0. The length to width ratios are 2.5, 2.3, 3.0, 5.5, 4.3, 4.3, 3.8, 3.0, 3.0, 3.0, and 3.8. The female pronotum is almost evenly convex, but, upon close examination, two semicircular, very shallow depressions are noticeable, one on either side of the meson, near the mid-length of the disc. The front trochanter lacks a spine; the front femora are not unusually enlarged, but instead are more slender than the hind femora and only slightly broader than the middle femora; the pro- and metatibiae are similar to those of the middle legs; and the hind femora are not abnormally enlarged. Tiny tibial spurs are present on all legs (on only the middle legs of males). In the front legs of females, the basitarsus is slightly longer than and about as wide as the second tarsomere. In the middle legs, the basitarsus is 1.5 times longer than and the same width as the second tarsomere. The tarsal setation is the same in all three pairs of legs, the basitarsi of the front and middle legs are lacking adhesive pads. The posterior margin of last ventrite is entire (Fig. 6F).

#### Female genitalia.

The bursa copulatrix is adorned with a carina and sclerotized patches (Fig. 6E). The spermatheca is bent distally, without distinct separation of the receptacle and pump (Fig. 6A, B); the maximum width is 0.04 mm. The vaginal palpi are bifurcate in the distal third (Fig. 6C), setose apically, widest basally and apically, and with a maximum width of 0.08 mm. The tignum is 1.18 mm long; the distal half has a membranous expansion, bearing setae (Fig. 6D).

#### Holotype.

“Ecuador:GuayasProv. \ Salanguillo 90 m. \ 19 FEB 1987 \ K. A. Johnson colr.” [1°58'S, 80°34'W, coordinates estimated, not included on label] (male, BYUC).

#### Paratypes.

Same data as holotype (4 females, BYUC; 1 female, QCAZ; 1 male, 1 female, TIC); same data as holotype, except 28 FEB 1987 (1 male, BYUC); “Ecuador, GuayasProv. \ SanAntonio, sealevel \ 3 km.S. Manglaralto \ 14 Feb 1987 \ K. A. Johnson colr.” [1°47'S, 79°32'W, coordinates estimated, not included on label] (1 male, QCAZ).

#### Etymology.

The species epithet, *fortis*, is Latin for strong, mighty, or powerful. It is in reference to the enormous front femora of the male.

#### Comments.

**Figure 7.**
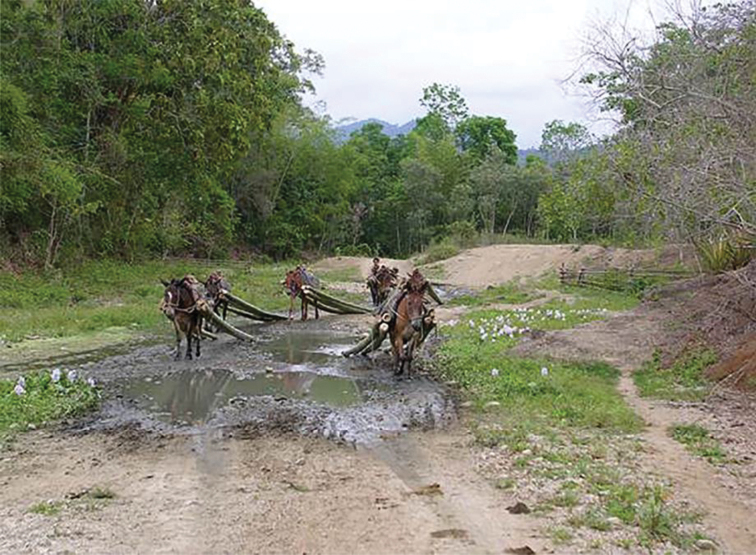
Area in Ecuador, near type locality of *Prathapanius fortis* sp. nov. (photograph by R. Wills Flowers).

This species occurs in a seasonally dry forest, near sea level, near the Pacific coast of Ecuador (Fig. 7). Because of the climate, the region has low biodiversity but high levels of endemism (Borchsenius 1997).

In addition to the remarkable modifications of the male front legs, the male genitalia are also extraordinary, with fin-like structures on both the median lobe and tegmen. Before the soft tissues were removed, the basal portion of the aedeagus was heavily surrounded by muscle tissue, somewhat similar to the condition found in the subfamilies Eumolpinae and Cryptocephalinae.

## Supplementary Material

XML Treatment for Prathapanius

XML Treatment for Prathapanius fortis

## References

[B1] Borchsenius F (1997) Patterns of plant species endemism in Ecuador. Biodiversity and Conservation 6: 379–399. 10.1023/A:1018312724137

[B2] Derunkov A Prado LR Tishechkin AK Konstantinov AS (2015) New species of *Diabrotica* Chevrolat (Coleoptera: Chrysomelidae: Galerucinae) and a key to *Diabrotica* and related genera: results of a synopsis of North and Central American *Diabrotica* species. Journal of Insect Biodiversity 3(2): 1–55. 10.12976/jib/2015.3.2

[B3] Duckett CN Gillespie JJ Kjer KM (2004) Relationships among the subfamilies of Chrysomelidae inferred from small subunit ribosomal DNA and morphology, with special emphasis on the relationship among the flea beetles and the Galerucinae. In: Jolivet P Santiago-Blay JA Schmitt M (Eds) New Developments on the Biology of Chrysomelidae.SPB Publishing, The Hague, 3–18.

[B4] Gillespie JJ Kjer KM Duckett CN Tallamy DW (2003) Convergent evolution of cucurbitacin feeding in spatially isolated rootworm taxa (Coleoptera: Chrysomelidae; Galerucinae, Luperini). Molecular Phylogenetics and Evolution 29(1): 161–175. 10.1016/S1055-7903(03)00256-2 12967617

[B5] Gillespie JJ Kjer KM Riley EG Tallamy DW (2004) The evolution of cucurbitacin pharmacophagy in rootworms: insight from Luperini paraphyly. In: Jolivet P Santiago-Blay JA Schmitt M (Eds) New Developments on the Biology of Chrysomelidae.SPB Publishing, The Hague, 37–57.

[B6] Gillespie JJ Tallamy DW Riley EG Cognato AI (2008) Molecular phylogeny of rootworms and related galerucine beetles (Coleoptera: Chrysomelidae). Zoologica Scripta 37: 195–222. 10.1111/j.1463-6409.2007.00320.x

[B7] Konstantinov AS (1998) Revision of the Palearctic species of *Aphthona* Chevrolat and cladistic classification of the Aphthonini (Coleoptera: Chrysomelidae: Alticinae). Memoirs on Entomology, International 11: 1–429.

[B8] Löbl I Smetana A (2010) Catalogue of Palaearctic Coleoptera (Vol. 6), Chrysomeloidea. Apollo Books, Stenstrup, 924 pp 10.1163/9789004260917

[B9] Mohamedsaid SM Furth DG (2011) Secondary sexual characteristics in the Galerucinae (*sensu stricto*) (Coleoptera: Chrysomelidae). International Scholarly Research Network, Zoology 2011: 1–60. 10.5402/2011/328670

[B10] Moura L (2003) Nova espécie de *Zischkaita* Bechyné e notas taxonômicas em Galerucini (Coleoptera, Chrysomelidae, Galerucinae). Revista Brasileira de Zoologia 20(4): 643–645. 10.1590/S0101-81752003000400014

[B11] Nie R Andújar C Gómez-Rodríguez C Bai M Xue H Tang M Yang C Tang P Yang X Vogler AP (2020) The phylogeny of leaf beetles (Chrysomelidae) inferred from mitochondrial genomes. Systematic Entomology 45(1): 188–204. 10.1111/syen.12387

[B12] Nie R Breeschoten T Timmermans MJTN Nadein K Xue H Bai M Huang Y Yang X Vogler AP (2018) The phylogeny of Galerucinae (Coleoptera: Chrysomelidae) and the performance of mitochondrial genomes in phylogenetic inference compared to nuclear rRNA genes. Cladistics 34: 113–130. 10.1111/cla.12196 34645082

[B13] Prado LR (2012) Review on the use of sexually dimorphic characters in the taxonomy of Diabroticites (Galerucinae, Luperini, Diabroticina). ZooKeys 332: 33–54. 10.3897/zookeys.332.4931 PMC3805318 24163580

[B14] Riley EG Clark SM Flowers RW Gilbert AJ (2002) Chrysomelidae Latreille 1802. In: Arnett RH Thomas MC Skelley PE Frank JH (Eds) American Beetles (Vol.2). Polyphaga: Scarabaeoidea through Curculionoidea. CRC Press, Boca Raton, 617–691.

[B15] Riley EG Clark SM Seeno TN (2003) Catalog of the leaf beetles of America north of Mexico (Coleoptera: Megalopodidae, Orsodacnidae and Chrysomelidae, excluding Bruchinae). Coleopterists Society, Special Publication 1: 1–290.

[B16] Rodrigues JMS Mermudes JRM (2015) Comparative morphology of the type-species of *Isotes* and *Synbrotica* (Coleoptera, Chrysomelidae, Galerucinae), with a new synonymy of species. Iheringia, Série Zoologia 105(4): 439–452. 10.1590/1678-476620151054439452

[B17] Seeno TN Wilcox JA (1982) Leaf beetle genera (Coleoptera: Chrysomelidae). Entomography 1: 1–221.

[B18] Smith EH (1979) Techniques for the dissection and mounting of the male (aedeagus) and female (spermatheca) genitalia of the Chrysomelidae. The Coleopterists Bulletin 33(1): 93–103. https://www.jstor.org/stable/4000169

[B19] Smith RF Lawrence JF (1967) Clarification of the status of the type specimens of Diabroticites (Coleoptera, Chrysomelidae, Galerucinae). University of California Publications in Entomology 45: 1–174.

[B20] Wilcox JA (1965) A synopsis of the North American Galerucinae (Coleoptera: Chrysomelidae). New York State Museum and Science Service Bulletin 400: 1–226.

[B21] Wilcox JA (1971) Chrysomelidae: Galerucinae. Oidini, Galerucini, Metacyclini, Sermylini. Coleopterorum Catalogus, Supplementa. Pars 78, Fasc. 1, (Editio Seconda). W. Junk, ‘s-Gravenhage, 220 pp.

[B22] Wilcox JA (1972) Chrysomelidae: Galerucinae Luperini: Aulacophorina, Diabroticina. Coleopterorum Catalogus, Supplementa. Pars 78, Fasc. 2, (Editio Seconda). W. Junk, ‘s-Gravenhage, 296–431.

[B23] Wilcox JA (1973) Chrysomelidae: Galerucinae Luperini: Luperina. Coleopterorum Catalogus, Supplementa. Pars 78, Fasc. 3, (Editio Seconda). W. Junk, ‘s-Gravenhage, 433–664.

